# Photodynamic priming enhances anti-PD1 therapy responses in patient-derived pancreatic cancer organoid immune cocultures

**DOI:** 10.3389/fimmu.2026.1805948

**Published:** 2026-08-11

**Authors:** Pushpamali De Silva, Demi Wekking, Joanna Joeun Choe, Madeline D. Kidd, Josie L. Pearce, Dario Missael Rocha Castellanos, Piotr Zelga, Russell Jenkins, Kenneth K. Wang, Vinay Chandrasekhara, Edward V. Maytin, Andrew Scott Liss, Tayyaba Hasan

**Affiliations:** 1 Wellman Center for Photomedicine, Massachusetts General Hospital and Harvard Medical School, Boston, MA, United States; 2 Amsterdam University Medical Center (UMC), Academic Medical Centre, University of Amsterdam, Amsterdam, Netherlands; 3 Rush University Medical College, Rush University, Chicago, IL, United States; 4 Yale University School of Medicine, New Haven, CT, United States; 5 Stanford Law School, Stanford, CA, United States; 6 Department of Surgery, Massachusetts General Hospital, and Harvard Medical School, Boston, MA, United States; 7 Massachusetts General Hospital Cancer Center and Harvard Medical School, Boston, MA, United States; 8 Laboratory of Pharmacology and Harvard Medical School, Boston, MA, United States; 9 Division of Gastroenterology and Hepatology, Mayo Clinic, Rochester, MN, United States; 10 Department of Biomedical Engineering, Lerner Research Institute, Cleveland Clinic, Cleveland, OH, United States; 11 Harvard-Massachusetts Institute of Technology (MIT) Health Sciences and Technology, Cambridge, MA, United States

**Keywords:** adaptive immunity, immune checkpoint blockade, immunogenic cell death, photodynamic therapy, tumor resistance mechanisms

## Abstract

Photodynamic priming (PDP), a fallout of photodynamic therapy, transiently modulates the tumor microenvironment (TME), enhances therapeutic susceptibility, and promotes immunogenic cell death through the release of damage-associated molecular patterns (DAMPs). Pancreatic ductal adenocarcinoma (PDAC) remains non-responsive to current therapies with a desmoplastic and immunosuppressive TME that limits drug delivery and blunts responses to immune checkpoint blockade. We investigated whether PDP could enhance anti-PD1 therapy responses in PDAC using patient-derived organoids (PDOs). PDOs were treated with Visudyne and red light (25, 75, and 100 J/cm²), followed by assessment of cytotoxicity, DAMP expression (HSP60, calreticulin, HMGB1), and transcriptomic changes. Monocyte-derived dendritic cells (mDCs) from healthy donors were cocultured with PDP-treated (25 J/cm^2^) PDOs, then with matched naïve T cells. mDC and T cell activation markers were analyzed. Pembrolizumab (anti-PD1) was added to PDO-mDC-T cell cocultures to evaluate combined effects on PDO viability and T cell activation. PDP induced dose-dependent cytotoxicity and upregulated DAMPs. Gene profiling in PDOs showed increased *IFN-γ*, *TNF-α*, and *CXCL12*, with reduced *PD-L1*, *TGF-β1*, and *FOXP1* expression. mDCs exposed to PDP-treated PDOs upregulated CD40, CD86, and MHC-II, driving activation of CD4^+^ and CD8^+^ T cells, evidenced by elevated PD1 expression. Addition of pembrolizumab further decreased PDO viability and amplified effector cytokines (*IFN-γ*, *TNF-α*, *FASLG*, *granzyme* B, *IL-2*, *IL-21*). PDP was associated with modulation of the PDAC TME toward a more immunogenic phenotype by enhancing tumor immunogenicity, activating dendritic cells and T cells, and potentiating PD1 blockade. These findings provide mechanistic support for further preclinical and clinical evaluation of PDP combined with checkpoint inhibition in PDAC.

## Graphical Abstract

**Figure:**
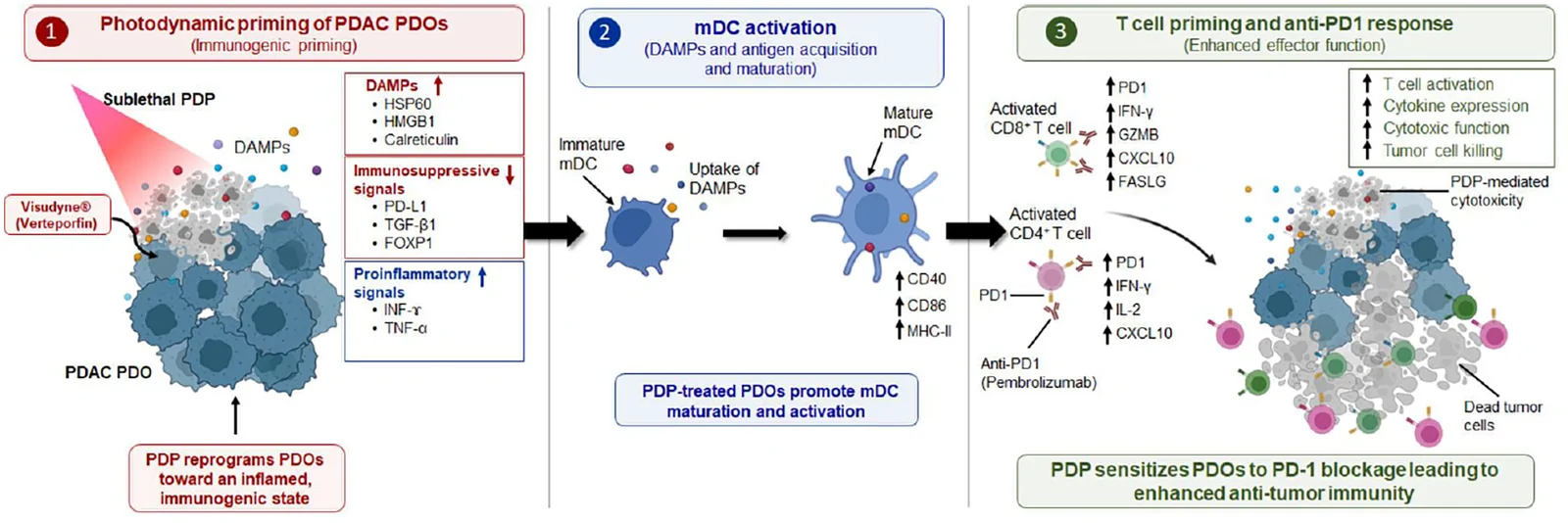
Sublethal photodynamic priming (PDP) of pancreatic ductal adenocarcinoma (PDAC) patient-derived organoids (PDOs) using Visudyne® (verteporfin) induces immunogenic remodeling of the tumor microenvironment characterized by increased release of damage-associated molecular patterns (DAMPs), including HSP60, HMGB1, and calreticulin, together with enhanced inflammatory signaling (IFNγ and TNFα) and reduced expression of immunosuppressive mediators (PD-L1, TGF-β1, and FOXP1). PDP-treated PDOs promote activation and maturation of immature monocyte-derived dendritic cells (mDCs), as evidenced by increased CD40, CD86, and MHC-II expression. Activated mDCs subsequently enhance CD4⁺ and CD8⁺ T cell activation-associated responses and effector function, leading to increased expression of anti-tumor mediators including IFN-γ, GZMB, IL-2, CXCL10, and FASL. In combination with anti-PD1 therapy (pembrolizumab), PDP sensitizes PDAC PDOs to immune checkpoint blockade, resulting in enhanced tumor control within the PDO coculture system. Created with BioRender.com.

## 1 Introduction

Pancreatic ductal adenocarcinoma (PDAC) is one of the most aggressive and lethal forms of cancer, characterized by a poor prognosis and limited treatment options (1). Despite advancements in cancer therapeutics, PDAC remains a formidable challenge due to its highly desmoplastic stroma and immunosuppressive tumor microenvironment (TME). The dense stroma not only acts as a physical barrier that impedes the delivery of therapeutic agents but also plays a crucial role in fostering an immunosuppressive milieu that protects the tumor from the host’s immune response (2–4). This immune exclusion largely explains the failure of immunotherapies in PDAC, with success rates confined to a small subset (1-3%) of patients whose tumors exhibit high microsatellite instability (3, 5, 6). These limitations underscore the urgent need for innovative combination strategies aimed at overcoming these barriers and enhancing treatment efficacy.

In the past decade, one of the encouraging treatment options for PDAC was photodynamic therapy (PDT) that combines light, a photosensitizer, and oxygen to induce localized cytotoxic effects and vascular damage within tumors. PDT works by activating the photosensitizer with light of a specific wavelength, leading to the production of reactive molecular species (RMS) that cause direct cell damage and induce apoptosis (7–11). Beyond its zone of cytotoxic effects, PDT modulates the TME in a process known as photodynamic priming (PDP) (11, 12). PDP, a sublethal form of PDT, transiently modulates tumor-immune interactions and may enhance responsiveness to subsequent therapies. This outcome is achieved through several factors including induced-stromal and vascular permeability and the release of damage-associated molecular patterns (DAMPs), which act as potent inducers of immunogenic cell death (ICD) to stimulate an immune response against the tumor. Given the challenges posed by the TME in PDAC, leveraging the immune-modulatory effects of PDP could potentially enhance the response to immunotherapy in PDAC.

This possibility is substantiated by recent studies, including our own and those of others. PDP enhances T cell priming, leading to increased T cell-mediated tumor killing in a three-dimensional (3D) PDAC coculture model (13). In an *in vivo* KPC mouse model, PDP was shown to modulate tumor stromal components, enhancing immune cell infiltration (14). The same study demonstrated that PDP induces both local and systemic immune responses. PDP significantly alters desmoplastic stromal components (12, 15–17), enabling more potent and sustained anti-tumor effects from chemotherapy (16, 18–20), even at reduced doses (17), improving tolerability in immunodeficient PDAC mouse models. Other studies have also shown that combining PDT with immunotherapies has yielded promising results in tumor regression in various solid tumor preclinical models (10, 21). Additionally, ongoing clinical studies highlight promising results for PDP as a minimally invasive, ablative therapy for advanced PDAC (22–24). Collectively, these findings suggest that PDP could be an ideal partner for immunotherapy in PDAC, a concept being evaluated in clinical trials such as the EUS-guided verteporfin PDT trial in locally advanced pancreatic cancer (NCT06381154).

Patient-derived organoids (PDOs) have credibility as models for studying cancer therapeutics. In this study, we investigated the potential of PDP to enhance the efficacy of anti-PD1 (Pembrolizumab) therapy in PDAC PDOs. PDOs are 3D cell cultures derived from patient tumors that closely mimic the heterogeneity and complexity of the original tumor, providing a more accurate model for studying therapeutic responses (25). We used Visudyne as the photosensitizer and administered varying light doses to induce PDP. Our objective was to evaluate the impact of PDP on cytotoxicity, the expression of DAMPs, and the subsequent immune response in PDOs. Our findings suggest that PDT induces direct cytotoxic effects, while PDP promotes immune-modulatory changes within PDO-based immune cocultures that are associated with enhanced anti-PD1 therapy responses. This combination therapy approach warrants further investigation as a potential strategy to improve outcomes in patients with PDAC, addressing the urgent need for more effective treatment options in this lethal cancer. Importantly, insights gained from PDO-based mechanistic studies may inform and refine the design of ongoing clinical trials, ultimately guiding more effective integration of PDP with immunotherapy in PDAC treatment.

## 2 Materials and methods

### 2.1 Tumor cell lines

MIA PaCa-2 (ATCC) PDAC tumor line, and Pancreatic Cancer-Associated Fibroblasts (PCAF) were cultured in Dulbecco’s Modified Eagle’s medium (DMEM). The PCAF cells were a kind gift from Dr. Diane Simeone. All media were supplemented with 10% heat inactivated Fetal Bovine Serum (FBS, Gibco), 100 U/mL penicillin, and 100 µg/mL streptomycin (Gibco) in T75 cell culture flasks (Corning) and maintained in a humidified CO_2_ atmosphere at 37 °C. All cells were confirmed negative for mycoplasma when tested using the MycoAlert Plus mycoplasma kit (Lonza), and this testing was done routinely.

### 2.2 Spheroid culture

Suspended 3D MIA PaCa-2 and PCAF cells were cultured in 96-well, round bottom ultralow attachment plates (Corning^®^ Costar^®^) at 37 °C. Tumor cells and PCAF cells were seeded at a density of 5,000 cells per well (1:1 ratio) for 48 hours to self-assemble into single 3D spheroids. This ratio is based on prior work from our group, supported by literature demonstrating that a higher ratio of PCAFs can rapidly outcompete PDAC cells.

### 2.3 Patient-derived organoid generation

Patient-derived organoids (PDOs) were generated from untreated PDAC patient tissues (n=7). PDAC patient tumor tissues were obtained for research and PDO generation from the Liss Laboratory at Massachusetts General Hospital (MGH), where a PDAC tumor bank with known genetic profiles is maintained, under Institutional Review Board-approved protocols (No. 2003P001289) with informed consent obtained from all patients. The study adheres to the Declaration of Helsinki regarding the use of human samples. PDO establishment was performed with slight modifications to previously explained protocols (13, 26–30) within 15 minutes upon receiving the samples. To establish organoids, tumor samples were minced into 1–2 mm pieces and incubated with 5 mg/mL collagenase XI (MilliporeSigma #C9407), 10 μg/mL DNase I (StemCell #07900), and 10 μM Y-27632 (MilliporeSigma #Y0503). The dissociated cells were embedded in domes of Corning^®^ Matrigel^®^ Basement Membrane Matrix in 24-well plates. After Matrigel solidification for 10 min at 37 °C, cells were cultured in PDO feeding media (**Supplementary Table 1**). Media was changed every 2–3 days and confluent organoids were passaged by incubating in Gentle Cell Dissociation Reagent (STEMCELL Technologies, #100-0485). Organoids were recovered by centrifugation and resuspended in Matrigel and maintained in PDO feeding media. During organoid establishment and passage, Y-27632 was included in the organoid growth media for 2–3 days until the media refreshment. Whenever necessary, organoids were dissociated into single cells via enzymatic digestion in TrypLE Express (Thermo Fisher Scientific) for 10 minutes at 37 °C. Well established and proliferative PDOs were able to grow in ultra-low attachment (ULA) plates without Matrigel for up to 14 days. Generated organoids were histopathologically assessed by comparing them with the original tumor tissue (**Supplementary Figure 1**).

All seven PDO lines were used for organoid establishment, expansion, and histopathological characterization. Different independently established PDO lines were subsequently used across experimental assays depending on organoid expansion capacity and compatibility with downstream coculture conditions. While all PDO lines were successfully maintained in Matrigel culture conditions, some lines did not reproducibly expand sufficiently in ULA conditions without Matrigel to generate the number of replicates required for downstream immune coculture experiments. Therefore, functional PDP, mDC, T cell, and anti-PD1 coculture studies were performed using PDO lines that demonstrated stable growth and reproducible expansion under the respective assay conditions.

### 2.4 Immune cell isolation from peripheral blood mononuclear cells

Healthy human buffy coats were purchased from BioIVT. The peripheral blood mononuclear cells (PBMC) fraction was isolated from buffy coats by Ficoll-Paque density gradient separation, which was then cryopreserved until later use. Naïve T cells or monocytes were separated from fresh or frozen PBMC using human Naïve Pan T Cell Isolation Kit or pan monocyte isolation kit (Miltenyi Biotec).

### 2.5 Generation of human monocyte-derived dendritic cells

Purified monocytes were cultured in the presence of GM-CSF (50 ng/ml) and IL-4 (50 ng/ml) (R&D Systems) in RPMI-1640 medium supplemented with 10% FBS, 1% Penicillin/Streptomycin, 1% Pyruvate, 1% L-glutamine, 1% HEPES, and 1% non-essential amino acids at 37 °C in humidified air containing 5% CO_2_. Cytokines were replenished in the cell culture on day 3, and within 6 days, monocytes had differentiated into mDCs with an immature phenotype (CD11C^+^/CD14^–^). On Day 6, quality control of the immature DC population was performed by flow cytometry. Lipopolysaccharide (LPS, from Escherichia coli 055: B5 strain 25 ng/ml, Sigma-Aldrich) were used as positive maturation controls.

### 2.6 PDP for PDAC PDOs

Based on our previous works (13, 31) 250 nM Liposomal Verteporfin (Visudyne^®^: Bausch + Lomb) was added to PDAC PDOs grown with Matrigel in 24 well-black walled plates or without Matrigel in 96 well-ULA plates when they reached 100-150 μm in diameter and incubated for 90 minutes (drug-light interval) in the dark at 37 °C. Thereafter, the 3D tumor models were washed three times with their culture medium followed by light activation (32, 33). The 0 J/cm² condition consisted of PDOs incubated with Visudyne and subjected to the identical wash procedure but without subsequent light exposure and served as the photosensitizer only/no light control. Red light (690 nm) was delivered at an irradiance of 150 mW/cm² through the plate bottom to achieve light doses of 25, 75, or 100 J/cm². Following PDP treatment, PDOs were cocultured with mDC or naïve T cells. For PDO and mDC cocultures, effector to target ratio of 1:1 was maintained while PDO to naïve T cells were cocultured at an effector to target ratio of 2:1.

### 2.7 RNA extraction and RT-qPCR

PDOs were separated from Matrigel by incubating in Gentle Cell Dissociation Reagent and recovered by centrifugation. CD4^+^ T cells or CD8^+^ T cells were FACS sorted from PDO-mDC-T cell cocultures. RNA was extracted using TRIzol Reagent (Invitrogen), and reverse transcribed using the High-Capacity RNA-to cDNA kit (Applied Biosystems). cDNAs from PDOs underwent 10 cycles of preamplification, while cDNAs from T cells were pre-amplified for 14 cycles using a pre-amplification buffer (Life Technologies) and a cocktail of primers. Cytotoxic molecules/ligand expressions (*IFN-γ*, *TNF-α*, *GZMB*, and *FASLG*), T cell cytokines/chemokines (*IL-2*, *IL-21*, *CXCL10* and *CXCL12*), and immunosuppressive molecules (*IL-10*, *TGF-β and FOXP1*) were analyzed. Primer sequences are listed in the **Supplementary Table 2**. Relative mRNA expression levels were normalized using the mean expression for two reference genes (*MLN51* and *EF1α)* with fold changes calculated using the 2^−ΔCt^ method. All RT-qPCR reactions were performed in technical duplicates.

### 2.8 Multicolor flow cytometry

*Hsp60, Calreticulin and HMGB1 detection:* Suspensions of PDOs were incubated with the manufacturers’ suggested dilution of fluorescently labeled primary monoclonal antibodies with relevant isotype controls (**Supplementary Table 3**). Intracellular labeling of HMGB1 was accomplished using fixed and permeabilized cells (prelabeled for membrane markers) with the BD Cytofix/Cytoperm™ Fixation/Permeabilization Solution Kit (BD Biosciences) following manufacturer’s protocol. Therefore, HMGB1 measurements reflect intracellular HMGB1-associated staining and do not assess extracellular HMGB1 release. Single cell suspensions were prepared from PDOs by mild enzymatic dissociation (TrypLE™ Express Enzyme, Thermo Fisher Scientific). *Detection of cell death and apoptosis in PDOs:* Suspensions of PDOs were incubated with the manufacturers’ suggested dilution of fluorescently labeled primary monoclonal antibodies including anti-CD45, anti-326 (EpCAM) prior to staining with Propidium Iodide (Biolegend) and Annexin V. Propidium Iodide and Annexin V-APC staining was done following manufacturer’s protocol (Biolegend). All antibody combinations used in this study including T cell and mDC marker flow panels are listed in the **Supplementary Table 3**).

### 2.9 Histopathology analysis

PDOs were separated from Matrigel by incubation in Gentle Cell Dissociation Reagent and collected by centrifugation. The PDOs were then wrapped in tissue paper (Bio-Wrap, Leica), folded, and inserted into an embedding cassette. The cells were fixed in 10% paraformaldehyde. Consecutive formalin-fixed, paraffin-embedded PDO sections (4 μm) were processed manually for conventional immunohistochemistry (IHC) or immunofluorescence assays, including Masson’s trichrome, PD-L1, Vimentin, αSMA, and H&E staining.

### 2.10 Protein isolation from PDOs and immunoblotting

3D tumor models were pooled (8 wells from each PDT-condition) 72 hours post-PDT. Proteins were extracted from 3D tumor models by lysis in Pierce RIPA lysis buffer (Thermo Scientific) supplemented with protease inhibitor (Thermo Scientific). Bicinchoninic acid assay (BCA) protein quantification (Thermo Scientific) was performed to quantify the protein concentration. Protein samples (20 μg) were loaded and separated on 4-20% polyacrylamide gel (Mini-PROTEAN TGX, Bio-Rad) at 100 V for 1–2 hours. Subsequently, the gel is blotted on a 0.45 μm transfer membrane (Thermo Scientific) for 1 hour at 100V. Next, the membranes were blocked with 5% BSA (Sigma) for 1 hour. After blocking, the membranes were incubated with primary antibody (listed in the **Supplementary Table 3**) in 2% BSA in 1X PBS (Corning) for 12–18 hours at 4 °C. Membranes were washed with 1X PBS and incubated for 2 hours with secondary antibodies labeled with horseradish peroxidase (ECL, 1:20000) in 1X PBS and 5% BSA.

### 2.11 Immune checkpoint inhibition

PDAC PDOs were treated with anti-PD1 (pembrolizumab, Merck: 125 μg/ml) (34) with InVivoMAb human IgG, BioXCell as an isotype control. The dose used is a 1:200 dilution of stock concentrations used clinically to correspond to reported peak plasma concentrations following administration of 10 mg/kg (FDA CDER application) (34). For anti-PD1 timing experiments, D0, D1, and D2 were defined relative to the start of T cell coculture rather than the timing of PDP treatment. Because naïve T cells were added 24 hours after initiation of PDO-mDC coculture with PDP-treated PDOs, anti-PD1 administration at D0, D1, and D2 of T cell coculture corresponds approximately to 24, 48, and 72 hours post-PDP, respectively.

### 2.12 Statistical analysis

All results are presented as mean ± standard deviation (SD) or standard error of the mean (SEM), as indicated in the respective figure legends. Statistical analyses were performed using GraphPad Prism Version 10 (GraphPad Software Inc., La Jolla, CA, USA). Specific statistical tests used for each dataset are indicated in the corresponding figure legends. All reported p values were two-tailed unless otherwise specified. Statistical analyses were performed using one-way or two-way ANOVA with appropriate *post hoc* multiple comparisons tests. Statistical significance was defined as p < 0.05. Independent experiments refer to biologically independent experiments performed at different times using independently prepared PDO cultures and immune cell preparations derived from different healthy donors where applicable. All experiments were performed with at least two technical replicates, as indicated in the figure legends. Experiments were analyzed as performed, without *post-hoc* pooling or removal of datasets.

## 3 Results

### 3.1 PDP enhances light-dose dependent photo-killing and the expression of DAMPs in PDOs

**Figure 1:**
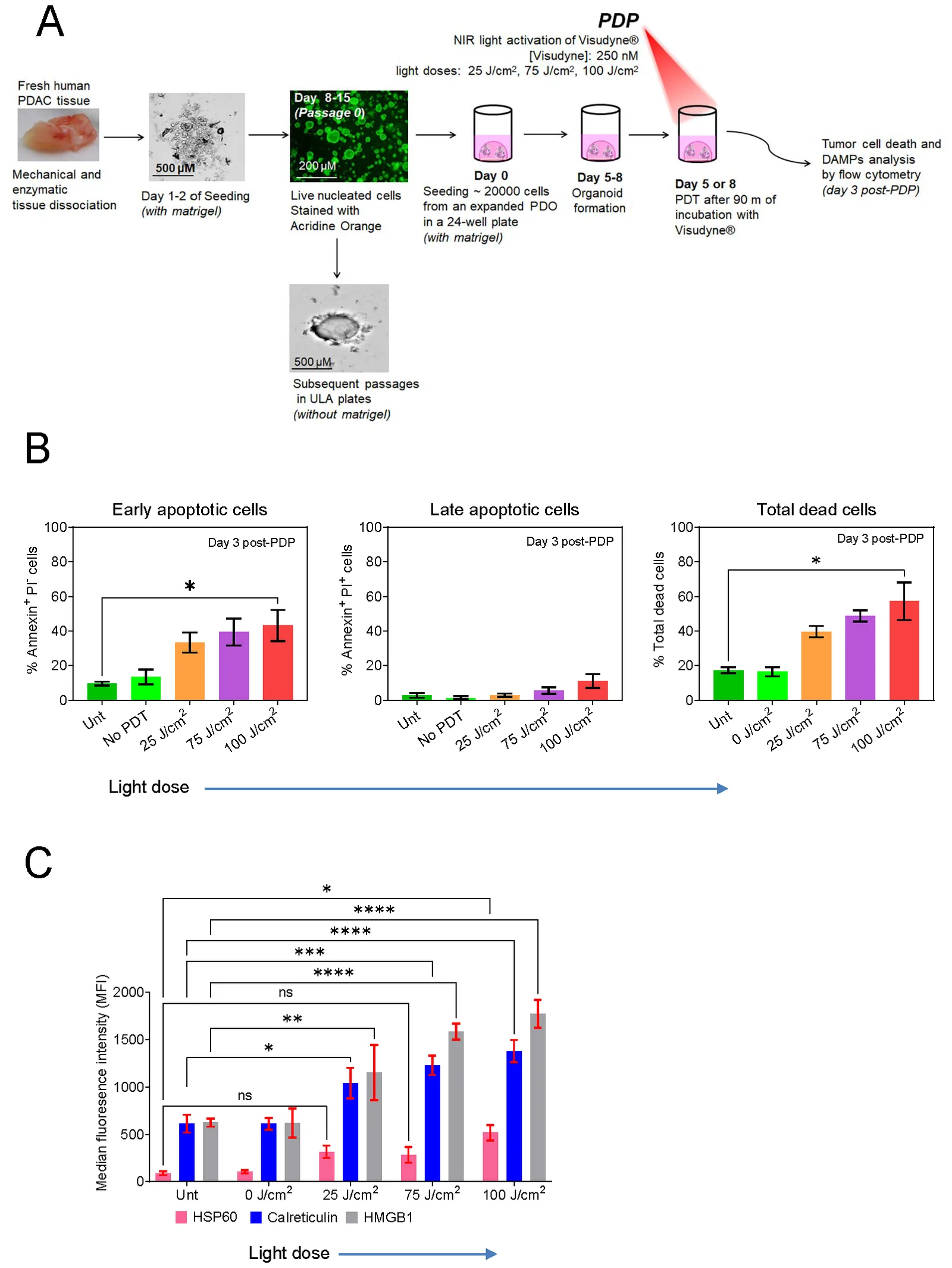
Phototoxicity and PDP-induced immunogenic cell death (ICD) in PDAC patient-derived organoids. **(A)** Schematic experiment plan for PDAC patient-derived organoid (PDO) establishment. Once expanded in Matrigel domes, PDOs were able to culture without Matrigel in ultra-low adhesion (ULA) plates. **(B)** Tumor cell death and **(C)** PDP-induced ICD markers (DAMPs: HSP60, Calreticulin, HMGB1) in PDOs were analyzed by flow cytometry (13) at day 3 post-PDP. The NIR photodynamic activation regimen used was 690 nm light irradiation with 25, 75 or 100 J/cm^2^ at 150 mW/cm^2^. Visudyne^®^ used as the photosensitizer at 250 nM. The percentage of early and late apoptotic cells and total dead cells (sum of early apoptotic, late apoptotic and necrotic cells) in PDOs following treatment with different doses of PDP were determined. Untreated (Unt) PDOs and those incubated with Visudyne and exposed to 0 J/cm² light served as controls. Data in **(B, C)** are presented as mean ± SEM of pooled three independent experiments from one PDAC PDO line performed in technical duplicates. Significance was calculated by a one-way ANOVA and Dunnett’s multiple comparisons test. Asterisks denote statistical significance (*P < 0.05; **P < 0.01; ***P < 0.001, ****P < 0.0001).

The schematic in **Figure 1A** outlines the experimental procedure used to evaluate the effects of PDP on PDAC PDOs. Tumor cell death was analyzed three days post-PDP treatment using flow cytometry The flow cytometry gating strategy was performed as previously described (13). The data reveal a light-dose dependent cytotoxicity and significant increase in cell death at 100 J/cm² (**Figure 1B**). Most dying tumor cells exhibited an early apoptotic phenotype at the analyzed time point. This could be due to the short endpoint in our assays (day 3 post-PDP) where the PDOs had just begun to die post-PDP. The effects of various doses of PDP on expression of DAMPs, including HSP60, calreticulin, and HMGB1, in PDOs were also evaluated (**Figure 1C**). The median fluorescence intensity (MFI) of each DAMP was measured by flow cytometry using a gating strategy similar to that previously described (13). Untreated PDOs and those exposed to 0 J/cm² light showed no significant induction of DAMPs. In contrast, treatment with 25 J/cm², 75 J/cm^2^ or 100 J/cm^2^ PDP resulted in a significant increase in HSP60, Calreticulin, and HMGB1 expression in a light-dose dependent manner. Notably, the 100 J/cm² dose induced the highest levels of DAMP expression, indicating that higher doses of PDP more effectively stimulate ICD markers. These results collectively demonstrate that PDP can induce dose-dependent tumor cell death and significantly upregulate the expression of key DAMPs in PDOs, suggesting its potential to enhance the immunogenicity of the TME in PDAC.

### 3.2 PDP-induced photomodulation of tumor immunogenicity in PDOs

**Figure 2:**
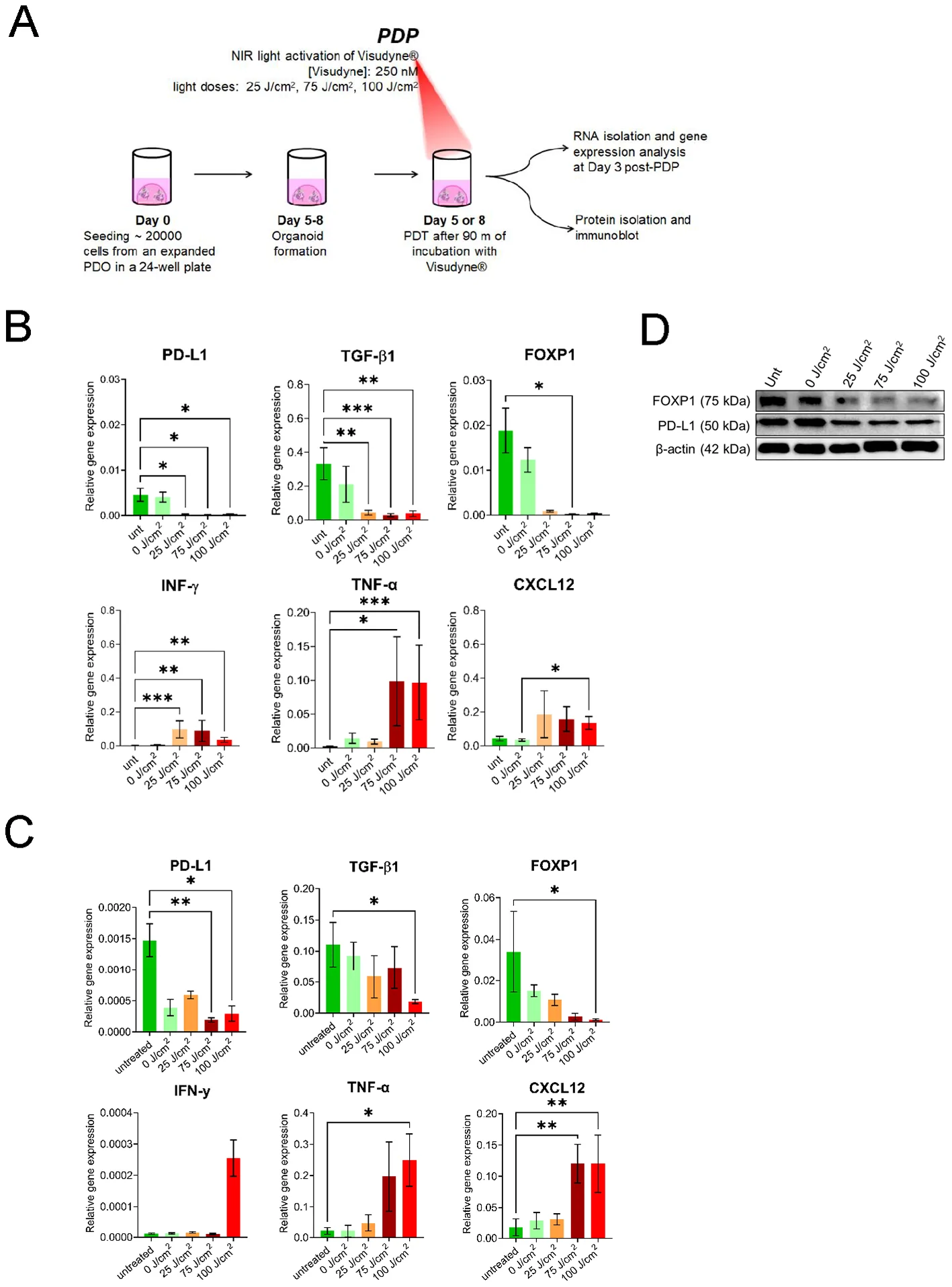
PDP modulates pro and anti-inflammatory factors in PDOs. **(A)** Schematic experimental workflow. PDAC PDOs expanded in Matrigel culture conditions were treated with PDP, followed by RNA and protein isolation for downstream RT-qPCR and immunoblot analyses. PDP-induced modulations in oncogenic and immune-associated gene expression profiles in **(B)** PDOs and **(C)** MIA PaCa-2 and PCAF spheroids. Oncogenic genes: *PD-L1*, *TGF-β1*, and *FOXP1* and immune-modulatory genes: *IFN-γ*, *TNF-α*, and *CXCL12* were analyzed by RT-qPCR. **(D)** FOXP1 and PD-L1 protein level confirmations in PDOs using immunoblotting. Data in **(B, C)** are presented as mean ± SEM of ≥3 biologically independent experiments performed using independently prepared PDO cultures and conducted in duplicates. The NIR photodynamic activation regimen used was 690 nm light irradiation with 25, 75 or 100 J/cm^2^ at 150 mW/cm^2^. Visudyne^®^ used as the photosensitizer at 250 nM. Untreated (Unt) PDOs and those incubated with Visudyne and exposed to 0 J/cm² light served as controls. Significance was calculated by a one-way ANOVA and Dunnett’s multiple comparisons test. Asterisks denote statistical significance (*P < 0.05; **P < 0.01; ***P < 0.001).

To understand PDP modulation of tumor intrinsic components, PDOs were exposed to varied light-doses (25 J/cm², 75 J/cm², and 100 J/cm²). RNA and protein were isolated for gene expression analysis and immunoblotting 3 days post-PDP (**Figure 2A**). There were significant decreases in protumor factors such as *PD-L1*, *FOXP1*, and *TGF-β1* expression with PDP doses of 25 J/cm², 75 J/cm², and 100 J/cm² compared to untreated control (**Figure 2B**). In contrast, there were increases in the proinflammatory cytokines IFN-γ and TNF-α, which are associated with anti-tumor immune responses, as well as CXCL12, a chemokine involved in immune cell trafficking and regulation of the TME, at different PDP doses. These findings were validated in MIA PaCa2-PCAF tumor spheroid cultures (**Figure 2C**), which similarly showed downregulation of *PD-L1*, *FOXP1*, and *TGF-β1*, and upregulation of *IFN-γ*, *TNF-α*, and *CXCL12*. Additionally, immunoblot analysis confirms the decrease of PD-L1 and FOXP1 proteins in PDOs treated with 25 J/cm², 75 J/cm², and 100 J/cm² doses of PDP compared to the untreated control (**Figure 2D**). These findings suggest that PDP treatment modulates both oncogenic factors and proinflammatory genes in PDOs, indicating potential therapeutic benefits in enhancing tumor immunogenicity.

### 3.3 Induction of mDC maturation in PDO cocultures treated with low-dose PDP

**Figure 3:**
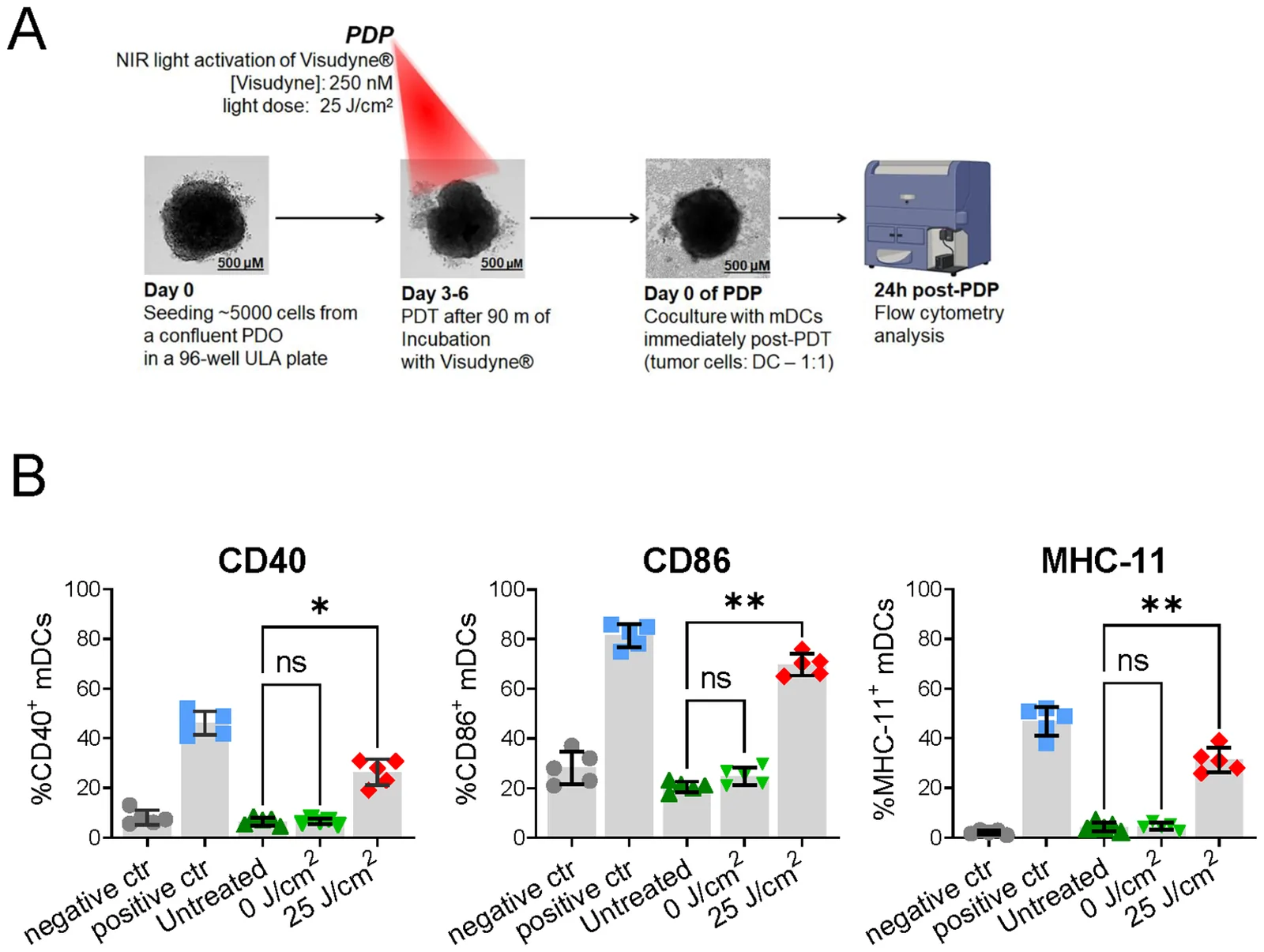
PDP induces the maturation of monocyte-derived dendritic cells (mDCs). **(A)** Schematic experimental plan. Briefly, PDOs were treated with low-dose PDT (250 nM of Visudyne; 25 J/cm^2^), then mDCs were added to an effector to target ratio of 1:1 and incubated for 24 hours. Each point denotes a different healthy donor-derived mDC. **(B)** Expression of CD40, CD86, and MHC-II maturation and activation markers was measured by flow cytometry. Data are presented as mean ± SD from five biologically independent experiments performed using five independent PDO lines and mDCs derived from five different healthy donors, with each experiment performed in technical duplicates. Each point represents an individual healthy donor and corresponds to the mean of duplicate measurements. Negative ctr, unstimulated mDCs; Positive ctr, LPS-stimulated mDCs; Untreated, mDCs cocultured with untreated PDOs; 0 J/cm^2^, mDCs cocultured with PDOs incubated with Visudyne but not exposed to light; 25 J/cm^2^, mDCs cocultured with PDOs incubated with Visudyne and exposed to 25 J/cm^2^ light. Significance was calculated by a one-way ANOVA and Dunnett’s multiple comparisons test. Asterisks denote statistical significance (*P < 0.05; **P < 0.01).

We investigated the maturation of monocyte-derived dendritic cells (mDCs) when cocultured with PDAC PDOs following PDP (**Figure 3A**). PDOs were initially treated with a low dose of PDT, involving the incubation with 250 nM Visudyne for 90 minutes and exposure to a light dose of 25 J/cm². Subsequently, mDCs were added to the PDOs and incubated for 24 hours. mDCs were added after completion of PDP treatment and were therefore not present during the Visudyne photoactivation. The maturation of these mDCs was assessed by evaluating the expression levels of CD40, CD86, and MHC-II using multicolor flow cytometry 24 hours after PDP. The expression levels of CD40, CD86, and MHC-II were measured across various experimental groups, including negative control (unstimulated mDCs), positive control (LPS-stimulated mDCs), untreated (mDCs cocultured with untreated PDOs), 0 J/cm² (mDCs cocultured with PDOs incubated with Visudyne but not exposed to light), and 25 J/cm² (mDCs cocultured with PDOs incubated with Visudyne and exposed to 25 J/cm² light). The data reveal significant increases in CD40, CD86, and MHC-II expression in mDCs cocultured with PDOs treated with 25 J/cm² light compared to the untreated and 0 J/cm² groups (**Figure 3B**). This upregulation of mDC maturation and activation markers indicates that the mDCs underwent maturation upon exposure to low-dose PDP. These findings suggest that, in addition to inducing tumor cell death, PDP may modulate the tumor immune microenvironment in a manner that enhances mDC functionality and could prime subsequent T cell activation.

### 3.4 Low-dose PDP-mediated induction of T cell priming in PDOs

**Figure 4:**
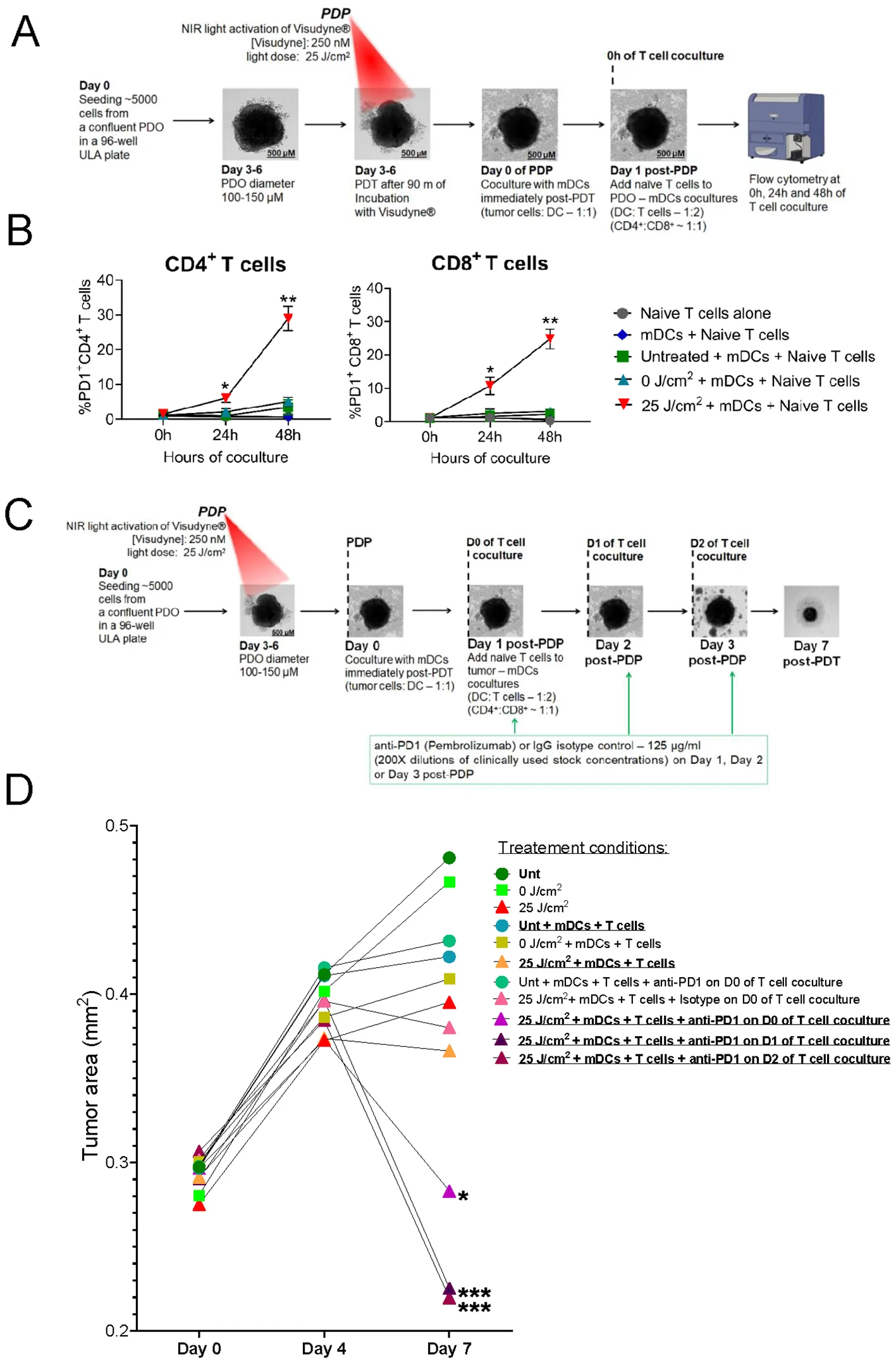
PDP-mediated PD1 expression in T cells and anti-PD1 therapy response in PDO cocultures. **(A)** Schematic experiment plan for PDO-mDC-naïve T cell coculture. **(B)** PD1 expression in CD4^+^ and CD8^+^ T cells measured by flow cytometry at 0 h, 24 h and 48 h of initial T cell coculture. **(C)** Schematic treatment plan for PDP + anti-PD1 combination treatments in PDOs. **(D)** PDP and anti-PD1 therapy responses in PDOs. *Unt*, untreated PDOs; *0 J/cm^2^*, PDOs incubated with Visudyne; *25 J/cm^2^*, PDOs incubated with Visudyne and treated with a light dose of 25 J/cm^2^; *Unt + mDC + T cells*, untreated PDO-mDC-T cell cocultures; *0 J/cm^2^ + mDC + T cells*, PDO-mDC-T cell cocultures with Visudyne, *25 J/cm^2^ + mDC + T cells*, PDP-mDC-T cell cocultures with Visudyne treated with a light dose of 25 J/cm^2^; *Unt + mDC + T cells + anti-PD1 on D0*, untreated PDO-mDC-T cell cocultures treated with anti-PD1 immediately after T cell coculture; *25 J/cm^2^ + mDC + T cells + Isotype*, PDP-mDCs-T cell cocultures treated with Visudyne and a light dose of 25 J/cm^2^ followed by IgG isotype control administered immediately after T cell addition (D0) to control for nonspecific antibody exposure; *25 J/cm^2^ + mDC + T cells + anti-PD1 on D0*, PDP-mDC-T cell cocultures with Visudyne and treated with a light dose of 25 J/cm^2^ and treated with anti-PD1 immediately after T cell coculture; *25 J/cm^2^ + mDC + T cells + anti-PD1 on D1*, PDP-mDC-T cell cocultures with Visudyne and treated with a light dose of 25 J/cm^2^ and treated with anti-PD1–24 h post T cell coculture; *25 J/cm^2^ + mDC + T cells + anti-PD1 on D2*, PDP-mDCs-T cell cocultures with Visudyne and treated with a light dose of 25 J/cm^2^ and treated with anti-PD1–48 h post T cell coculture. T cells were sorted on day 7 from the underlined treatment conditions to assess their activation. For anti-PD1 timing experiments, D0, D1, and D2 were defined relative to the start of T cell coculture rather than PDP treatment. Data in **(B)** are presented as mean ± SD from three biologically independent experiments performed using three independent PDO lines and naïve T cells derived from three different healthy donors, with each experiment performed in technical duplicates. Each point represents an individual healthy donor and corresponds to the mean of duplicate measurements. Significance was calculated by a two-way ANOVA and Tukey’s multiple comparisons test. Asterisks denote statistical significance (*P < 0.05; **P < 0.01). Data in **(D)** are presented as mean ± SEM from biologically independent experiments performed using five independent PDO lines and immune cell preparations from five different healthy donors, with each experiment performed in technical duplicates. Significance was calculated by a two-way ANOVA and Dunnett’s multiple comparisons test. Asterisks denote statistical significance (*P < 0.05; ***P < 0.001). Error bars are omitted for clarity of visualization.

Compelling evidence on the effects of PDP in enhancing T cell priming within PDO cocultures of PDAC is presented in **Figure 4**. As shown in the **Figure 4A** schematic experimental plan, PDOs were first cocultured with mDCs 24 hours post-PDP, followed by the addition of naïve T cells for a further 24–48 hours of coculture. The 24 hour interval prior to T cell addition was intentionally used to model an antigen acquisition and maturation phase, allowing mDCs to interact with PDP-treated tumor cells before naïve T cell exposure. The results demonstrate increased PD1 expression on both T cell subsets when cocultured with PDP-treated PDO-mDCs. At 24 and 48 hours of coculture, the percentage of PD1^+^CD4^+^ and PD1^+^CD8^+^ T cells was markedly higher in the 25 J/cm² light-treated groups compared to controls (**Figure 4B**). These findings suggest that PDP promotes early T cell-associated immune priming events within the coculture system. Although PD1 expression alone is not sufficient to define effective T cell activation, the observed increase in PD1^+^ CD4^+^ and CD8^+^ T cells is consistent with enhanced T cell-associated immune activation within the coculture model. Collectively, these findings highlight the potential of PDP to modulate the immunosuppressive PDAC tumor microenvironment toward a more immune-responsive state that may enhance responsiveness to anti-PD1 therapy.

### 3.5 Low-dose PDP and anti-PD1 combination therapy response in PDOs

Motivated by the above findings, we investigated the combined effect of PDP and anti-PD1 immunotherapy on the growth of PDOs. The detailed schematic of the experimental design is shown in **Figure 4C**, which includes treating PDOs with PDP, followed by coculturing with mDC and naïve T cells and then administering the anti-PD1 therapy (Pembrolizumab) at different time points, day 0 (D0), day 1 (D1), and day 2 (D2) of T cell coculture. The tumor area was determined by live imaging, which spanned 7 days post-PDP treatment. The data revealed a significant reduction in PDO growth at day 7 when treated with the combination of PDP and anti-PD1 therapy on D0, D1, and D2 of T cell coculture, compared to the controls and other single treatment groups (**Figure 4D**). Specifically, the combination of 25 J/cm² light-activated Visudyne with mDCs and T cells followed by anti-PD1 treatment on D1 and D2 of T cell coculture showed the most pronounced decrease in tumor growth. This significant decrease in tumor growth suggests that PDP enhances responsiveness to anti-PD1 therapy within the PDO-based coculture system. Combination therapy likely enhances the activation and efficacy of T cells within the PDOs, leading to improved immunotherapeutic outcomes. These findings underscore the potential of integrating PDP with immunotherapy as a promising strategy for treating PDAC, which is typically resistant to immunotherapy and other conventional therapies.

### 3.6 Low-dose PDP-anti-PD1 combination therapy induces T cell activity in PDOs

**Figure 5:**
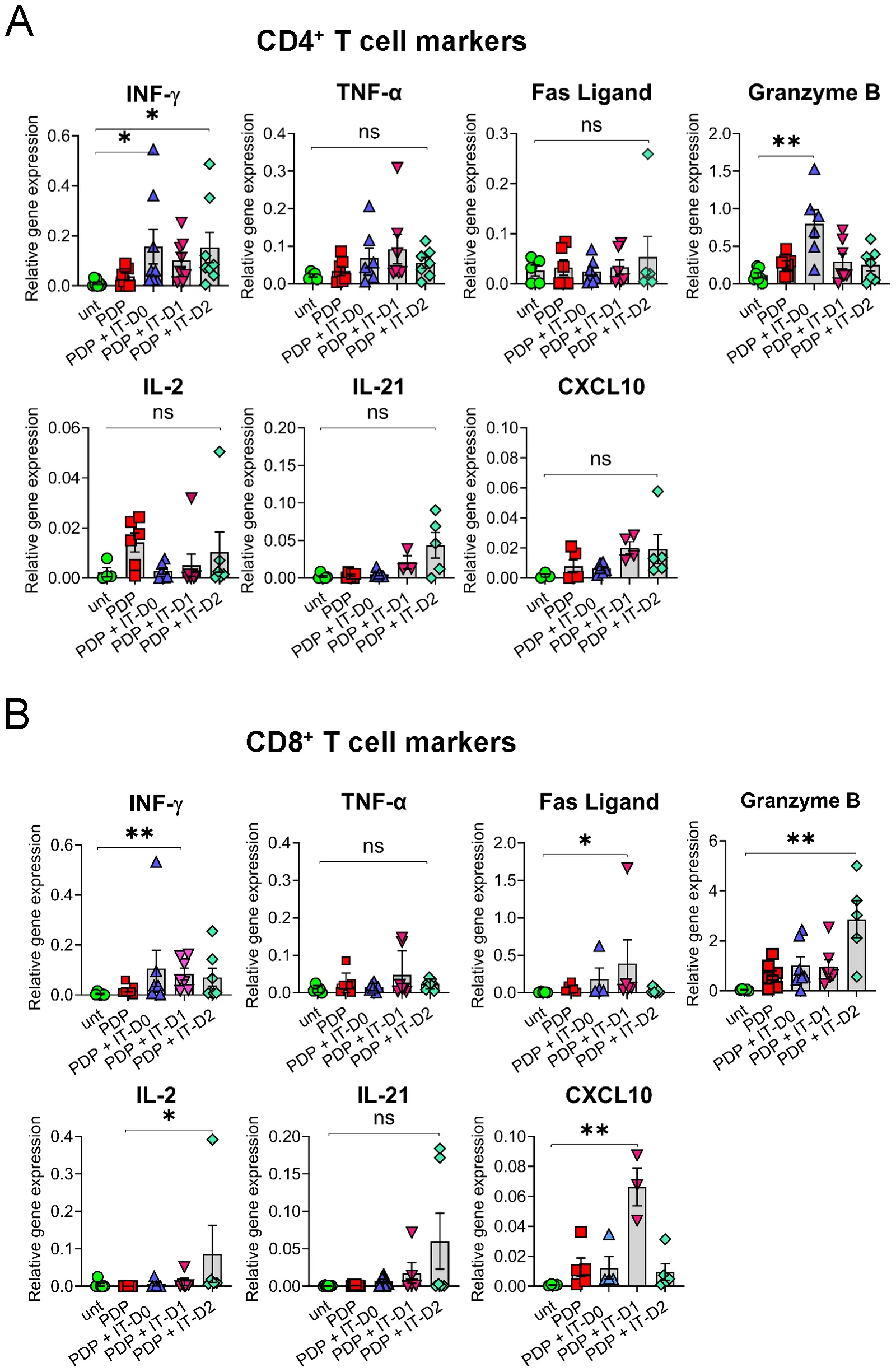
PDP-anti-PD1 combination therapy induces T cell activity in PDOs. CD4^+^ and CD8^+^ T cells were FACS sorted from the PDO-mDC-T cell cocultures that were used to analyze the therapy responses in **Figure 4D** (underlined conditions). Gene expression analysis was performed by RT-qPCR in sorted T cells to study T cell activation markers. **(A)** CD4^+^ T cell and **(B)** CD8^+^ T cell activation marker gene expression. In **(A, B)**, data are presented as mean ± SEM from >3 biologically independent experiments performed using independently prepared PDO cultures and T cell preparations from >3 different healthy donors, with each experiment performed in technical duplicates. Each point represents an individual healthy donor and corresponds to the mean of duplicate measurements. Significance was calculated by a one-way ANOVA and Dunn’s multiple comparisons test. Asterisks denote statistical significance (*P < 0.05; **P < 0.01). *Unt*, T cells sorted from untreated PDO-mDC-T cell cocultures; *PDP*, T cells sorted from PDP-mDC-T cell cocultures treated with a light dose of 25 J/cm^2^; *PDP+IT-D0*, T cells sorted from PDP plus anti-PD1 therapy on D0 of T cell coculture; *PDP+IT-D1*, T cells sorted from PDP plus anti-PD1 therapy on D1 of T cell coculture; *PDP+IT-D2*, T cells sorted from PDP plus anti-PD1 therapy on D2 of T cell coculture. *IT*, Immunotherapy.

The impact of low-dose PDP-anti-PD1 combination therapy on T cell activation was evaluated by analyzing the gene expression of key activation markers in CD4^+^ and CD8^+^ T cells sorted from selected cocultures (bold and underlined conditions in **Figure 4D**) on day 7. Treatment conditions included untreated PDO-mDC-T cell cocultures (Unt), PDP-treated (25 J/cm²) cocultures (PDP), and cocultures treated with PDP plus anti-PD1 therapy on various days of T cell coculture (PDP+IT-D0, PDP+IT-D1, PDP+IT-D2), as shown on **Figures 4C** and **D**. For CD4^+^ T cells, there was a significant increase in *IFN-γ* and *GZMB* (Granzyme B*)* expression in the PDP+IT-D0 compared to Unt (**Figure 5A**). No significant differences were observed in *TNF-α*, *FASLG* (Fas Ligand), *IL-2*, *IL-21*, and *CXCL10* expression across the different treatment groups. However, the PDP+IT-D2 group showed a significant increase in *IFN-γ* expression compared to other groups. In CD8^+^ T cells, *IFN-γ, FASLG*, and *CXCL10* expressions were significantly increased in the PDP+IT-D1 while GZMB and *IL-2* were upregulated in PDP+IT-D2 groups compared to untreated and PDP+IT-D0 groups (**Figure 5B**). *TNF-α* and *IL-21* expression did not show significant differences across the treatment groups. These findings indicate that PDP-anti-PD1 combination therapy significantly activates both CD4^+^ and CD8^+^ T cells, particularly enhancing the expression of *IFN-γ*, *GZMB*, *IL-2*, *FASLG*, and *CXCL10*. The activation kinetics varied depending on the timing of anti-PD1 administration, suggesting that the schedule of checkpoint blockade influences the magnitude and nature of the T cell response. The variation in activation based on the timing of anti-PD1 administration underscores the potential importance of treatment scheduling, an aspect that merits further investigation.

## 4 Discussion

Pancreatic ductal adenocarcinoma (PDAC) presents significant challenges due to its aggressive nature and resistance to conventional therapies. The dense stroma and immunosuppressive TME characteristic of PDAC contribute to poor therapeutic outcomes. This study aimed to investigate the potential of PDP to enhance the efficacy of anti-PD1 therapy (Pembrolizumab) in PDOs of PDAC. Our findings support the possibility that PDP can induce direct cytotoxic effects and modulate the TME toward a more immunogenic state, thereby improving the response to immunotherapy.

During PDP, RMS are generated when a photosensitizer is excited by light of a specific wavelength in the presence of oxygen. This process results in direct cytotoxic effects that target and kill tumor cells. In our study, the initial results demonstrated a dose-dependent cytotoxic effect of PDP on PDAC PDOs, where higher doses of light led to increased levels of cell death. However, despite these effects, we observed that by three days post-PDP treatment, complete eradication of PDOs was not achieved, even with the highest doses of PDP. This incomplete killing may be related to the particularly dense and compact structure of PDAC PDOs, which could limit the penetration and efficacy of the treatment. Another explanation could be that we only administered a single treatment of PDP. In addition, a dedicated light-only control was not included in all PDO-immune coculture assays. However, consistent with the established mechanism of PDT, biologic effects are primarily dependent on photoactivation of the photosensitizer, whereas light-only exposure at the fluence used here has generally shown minimal toxicity in prior studies (11, 13, 15, 35). In our prior Visudyne-mediated PDT/PDP studies performed under comparable irradiation conditions, light-only exposure similarly did not induce substantial cytotoxic effects. Nevertheless, future studies should include dedicated light-only controls across all functional coculture assays to formally exclude potential photosensitizer-independent light effects. Moreover, following PDP treatment, there was a significant upregulation of DAMP expression such as HSP60, calreticulin, and HMGB1. The increased presence of these DAMPs is crucial because they are involved in the process of ICD, which plays a vital role in activating the immune system to recognize and attack tumor cells. The induction of ICD is particularly noteworthy, as it enhances the body’s adaptive immune response and helps establish long-term immunological memory, which is essential for preventing tumor recurrence. PDP is recognized as a potent inducer of ICD, distinguishing it from many other anti-cancer therapies that do not effectively trigger this immune response (14, 36, 37). By inducing ICD, PDP not only directly damages and kills tumor cells but also modulates the TME to make it more conducive to an immune attack. This dual mechanism of tumor cell killing and immunological priming highlights the therapeutic potential of PDP. It suggests that PDP could represent a strategy not only for immediate tumor cell destruction but also for promoting longer-term anti-tumor immune responses.

Furthermore, PDP treatment modulated the expression of key oncogenic and immune-associated mediators in PDOs. We observed significant decreases in protumor markers such as *PD-L1*, *FOXP1*, and *TGF-β1*, while immune-associated cytokines like *IFN-γ*, *TNF-α*, and *CXCL12* were upregulated. This shift in the cytokine milieu suggests that PDP may modulate the TME from an immunosuppressive state to an immunogenic one, potentially enhancing the effectiveness of subsequent immunotherapies. The decrease in PD-L1 expression in PDOs and tumor spheroid models aligned with our findings from *in vivo* PDAC studies (14). Interestingly, previous work demonstrated that PDP coupled with vitamin D3 receptor activation within fibroblasts increased intratumoral nanoliposomal irinotecan accumulation and suppressed protumorigenic CXCL12/CXCR7 crosstalk (17). In contrast, our study observed an upregulation of *CXCL12* in PDOs following PDP treatment. This difference could be due to several factors, such as the use of a different photosensitizer or tumor model. In this study, we used Visudyne as the photosensitizer and the anticancer effects of Visudyne-PDT have been evaluated in several clinical trials for metastatic PDAC (22, 23) (NCT03033225). These studies demonstrated the safety and efficacy of Visudyne-PDT, highlighting its promise as a minimally invasive ablative therapy. Furthermore, Visudyne-PDT is currently being tested in clinical trials for prostate cancer (38) and metastatic breast cancer (39). Together, these findings suggest that Visudyne-based PDP may modulate tumor-immune interactions and warrants further investigation as a potential adjunct to immunotherapy in PDAC and other solid tumors.

A key aspect of this study was examining the maturation of mDCs in response to low-dose PDP-treated PDOs. The results revealed significant upregulation of maturation markers CD40, CD86, and MHC-II in mDCs cocultured with PDP-treated PDOs. The increased expression of CD40, a critical marker of mDC maturation, suggests that PDP effectively drives this process (40, 41). Similarly, the elevated levels of CD86 indicate a heightened activation state in mDCs, which is crucial for initiating immune responses. As a co-stimulatory molecule, CD86 is essential for T cell activation, highlighting the enhanced functional capability of mDCs following PDP treatment. Moreover, the upregulation of MHC-II reflects an improved ability of mDCs to present antigens and activate T cells, further emphasizing PDP’s potential to boost anti-tumor immunity. Collectively, these findings demonstrate that PDP treatment induces robust maturation of mDCs, as evidenced by the enhanced expression of CD40, CD86, and MHC-II, thereby promoting a more effective immune response within the TME.

Our work also demonstrated the effects of PDP on T cell-associated immune priming within PDO cocultures. There was a notable increase in PD1 expression on both CD4^+^ and CD8^+^ T cells following PDP treatment. Although PD1 expression alone is not sufficient to define productive T cell activation, the combined increase in activation- and effector-associated marker expression together with enhanced responsiveness to anti-PD1 therapy supports PDP-associated enhancement of T cell-associated immune activation within the coculture system. The combination of PDP and anti-PD1 therapy showed a significant reduction in PDO growth compared to controls and single treatment groups. This suggests that PDP may sensitize the TME, making it more responsive to immunotherapy. The enhanced T cell-associated immune activation observed in the combination therapy further supports the potential of this approach to improve anti-tumor immune responses in PDAC. Finally, the study highlighted increased expression of key activation-associated markers such as *IFN-γ*, *GZMB*, *IL-2*, *FASLG*, and *CXCL10* in both CD4^+^ and CD8^+^ T cells following PDP-anti-PD1 combination therapy. Collectively, these findings are consistent with enhanced effector-associated T cell responses within the coculture system.

Although the PDO-immune coculture platform enabled controlled mechanistic evaluation of PDP-associated tumor-immune interactions within a human-based system, the model does not fully recapitulate the complex desmoplastic and immunosuppressive PDAC TME. In addition, immune cells used in this study were derived from healthy donors rather than matched autologous PDAC patients. While this approach enabled a controlled assessment of PDP-associated immune modulation, it may not fully capture patient-specific immune dysfunction and immunosuppressive features present in PDAC. Nevertheless, this platform provides a valuable mechanistic framework to study PDP-associated immune modulation in human PDAC. Future studies incorporating more complex *in vivo* or multicellular model systems will be important to further evaluate the translational relevance of these findings.

In summary, our findings show that PDT provides direct cytotoxic control of tumors, while PDP enhances immunogenicity and sensitizes PDAC to anti-PD1 therapy. Sublethal PDP dosing (~25 J/cm²) was associated with induction of ICD-associated responses, modulation of the TME, and enhanced immune activation within the PDO-based coculture system. The timing-dependent responses observed in this study suggest that the sequencing of PDP and anti-PD1 therapy may influence treatment outcomes. However, these findings should be considered mechanistic and hypothesis-generating and warrant further evaluation in more complex preclinical models and clinical studies. Induction of DAMPs, suppression of immunoregulatory mediators, and activation of IFN-associated T cell programs highlight candidate biomarkers for future translational investigation. Beyond the immediate necrotic effects of PDT, the downstream impact of PDP may involve immune-mediated clearance of residual tumor cells. Activated T cells generated within the PDP-associated immunogenic milieu may contribute to the elimination of residual tumor cells. What remains to be determined is whether tumor cells that withstand both light exposure and photosensitizer treatment are enriched for enhanced ICD-associated responses and whether these residual populations are particularly responsive to anti-PD1 therapy. Future studies comparing PDP with non-photodynamic cytotoxic therapies, including chemotherapy-based approaches, may help further define which immune-modulatory effects are specific to PDP versus more general treatment-induced responses. Future studies should determine whether a quantifiable relationship between direct cytotoxic killing and ICD-driven immune responses exists, which may help define the therapeutic balance and predict treatment outcomes not only in PDAC but also in other immunologically cold solid tumors.

## Data Availability

The original contributions presented in the study are included in the article/**Supplementary Material**, further inquiries can be directed to the corresponding author/s.

## Supplementary material

Supplementary Figure 1 Histopathological assessment of patient-derived organoids (PDOs). **(A, B)** PDOs were evaluated for histological similarity to the original tumor tissue. Consecutive 4 μm formalin-fixed, paraffin-embedded sections of PDOs were stained using Masson’s trichrome or subjected to immunofluorescence staining for Vimentin and αSMA. Organoid architecture and marker expression were compared to the corresponding primary tumor tissues. All staining procedures were performed manually. **(A)** Representative Masson’s trichrome staining of two PDAC tumor tissues (top panel: PDAC-1 and PDAC-2; scale bar: 500 µm), showing differential collagen content (blue), and their matched PDOs (bottom panel: PDO-1 and PDO-2; scale bar: 250 µm). **(B)** Immunofluorescence staining of Vimentin, Cytokeratin, and FAPα expression in PDAC tumor tissues (top panel: PDAC-3 and PDAC-4; scale bar: 500 µm) and their corresponding PDOs (bottom panel: PDO-3 and PDO-4; scale bar: 100 µm).
